# Genetic and Developmental Basis for Increased Leaf Thickness in the Arabidopsis Cvi Ecotype

**DOI:** 10.3389/fpls.2018.00322

**Published:** 2018-03-14

**Authors:** Viktoriya Coneva, Daniel H. Chitwood

**Affiliations:** Donald Danforth Plant Science Center, St. Louis, MO, United States

**Keywords:** leaf thickness, Ler × Cvi RILs, endopolyploidy, palisade mesophyll cell elongation, stress-growth tradeoff

## Abstract

Leaf thickness is a quantitative trait that is associated with the ability of plants to occupy dry, high irradiance environments. Despite its importance, leaf thickness has been difficult to measure reproducibly, which has impeded progress in understanding its genetic basis, and the associated anatomical mechanisms that pattern it. Here, we used a custom-built dual confocal profilometer device to measure leaf thickness in the Arabidopsis Ler × Cvi recombinant inbred line population and found statistical support for four quantitative trait loci (QTL) associated with this trait. We used publically available data for a suite of traits relating to flowering time and growth responses to light quality and show that three of the four leaf thickness QTL coincide with QTL for at least one of these traits. Using time course photography, we quantified the relative growth rate and the pace of rosette leaf initiation in the Ler and Cvi ecotypes. We found that Cvi rosettes grow slower than Ler, both in terms of the rate of leaf initiation and the overall rate of biomass accumulation. Collectively, these data suggest that leaf thickness is tightly linked with physiological status and may present a tradeoff between the ability to withstand stress and rapid vegetative growth. To understand the anatomical basis of leaf thickness, we compared cross-sections of Cvi and Ler leaves and show that Cvi palisade mesophyll cells elongate anisotropically contributing to leaf thickness. Flow cytometry of whole leaves show that endopolyploidy accompanies thicker leaves in Cvi. Overall, our data suggest that mechanistically, an altered schedule of cellular events affecting endopolyploidy and increasing palisade mesophyll cell length contribute to increase of leaf thickness in Cvi. Ultimately, knowledge of the genetic basis and developmental trajectory leaf thickness will inform the mechanisms by which natural selection acts to produce variation in this adaptive trait.

## Introduction

Understanding the developmental basis of evolutionary change in leaf shape presents several major challenges. Not only is leaf shape inherently multidimensional, thus necessitating multivariate methods for its quantification, but it also changes throughout the organ’s ontogeny in response to a complex interplay of genetic and environmental factors. Finally, adaptive evolutionary change in leaf shape operates on this high dimensional space encompassing multiple genetic, tissue mechanics, environmental, and ontogeny-specific factors (Langlade et al., 2005; Klingenberg, 2010; Merks et al., 2011; Baker et al., 2015; Fu et al., 2017). While leaf shape traits have been shown to have a heritable albeit polygenic basis (Langlade et al., 2005; Tian et al., 2011; Chitwood et al., 2013, 2014), they are also highly plastic to environmental cues such as temperature and moisture (Royer and Wilf, 2006; Peppe et al., 2011).

Despite the functional significance of leaf shape to environmental adaptation (Nicotra et al., 2011), however, few specific aspects of leaf shape have been linked directly to adaptive outcomes. Multivariate analyses have enabled the delineation of global (i.e., spanning taxonomic and biome groups) associations between biogeographic parameters, such as temperature, irradiance, and precipitation and specific leaf traits, which are deemed functional (Wright et al., 2004, 2017). Among these functional traits, leaf mass per area (LMA), the product of a leaf’s thickness and its density, is robustly associated with the ability of plants to survive arid, high-irradiance environments (Wright et al., 2004; Poorter et al., 2009).

Thick leaves can maintain water potential when water supply is low. A variety of morphological and anatomical features that vary with both phylogenetic context and the aridity of the habitat underlay this adaptive feature (Ogburn and Edwards, 2010). At the cellular level leaf thickness is broadly associated with increased cell size (Watson, 1942; Gibson, 1982; von Willert, 1992), which promotes water storage (Becker, 2007). Recent work in tomato showed that a specific leaf cell type, the palisade mesophyll, contributes to increased thickness in a desert-adapted wild species (Coneva et al., 2017). This finding is supported by allometric studies showing that leaf thickness scales specifically with the size of palisade mesophyll cells (Garnier and Laurent, 1994; Roderick et al., 1999; Sack and Frole, 2006; John et al., 2013). Studies exploring the functional physiology of palisade cell morphology show that increased cell height leads to improved uptake of carbon dioxide (CO_2_) into mesophyll cells, and improved photosynthesis in thick leaves (Oguchi et al., 2005; Terashima et al., 2011). The cylindrical shape of palisades has also been functionally linked to a more efficient distribution of light throughout the mesophyll (Brodersen et al., 2008; Brodersen and Vogelmann, 2010). At the organismal level, thicker leaves present a tradeoff between rapid growth versus drought and heat tolerance (Smith et al., 1997; Edwards et al., 2014). This idea is supported by global correlations between LMA, a proxy for leaf thickness, and habits associated with slower growth (Poorter et al., 2009).

Despite the importance of leaf thickness to plant physiology, little is known about the developmental manifestations of thick leaves, especially in plant families without succulent species. Apart from a few known mutants in Arabidopsis that produce thicker leaves, such as *angustifolia* and *rotundifolia3* (Tsuge et al., 1996), as well as *argonaute1*, *phantastica*, and *phabulosa* (which have aberrations in the polarity of cell elongation; Bohmert et al., 1998), our understanding of the genetic mechanisms by which natural selection may act to pattern quantitative variation in leaf thickness is poor. A transcriptomic experiment of developing leaves in two desert tomato (*S. pennellii*) introgression lines suggests that alterations in cell-cycle and endoreduplication events during early leaf development are at least partially responsible for the patterning of desert-adapted leaves in tomato (Coneva et al., 2017) thus connecting the rewiring of ontogenic networks to micro-evolutionary change in leaf thickness.

Since leaf thickness is defined on an eco-physiological (i.e., functional) rather than a taxonomic basis, it is necessary to investigate the genetic basis and general morphology of leaf thickness in multiple phylogenetic contexts with the ultimate goal of building an integrated framework of the developmental trajectory of this trait. This understanding will elucidate the mechanisms by which natural selection acts to produce variation in this adaptive trait. One approach to dissect the genetic loci that condition variation in leaf thickness is to employ quantitative genetic approaches to statistically associate leaf thickness with the genetic loci that regulate it. While such methods have been used to understand the genetic basis of abiotic stress responses in plants (reviewed in Collins et al., 2008), one of the challenges in addressing leaf thickness using quantitative genetic approaches is the fact that direct, precise, and reproducible measurements of this trait are technically difficult. Recently, we reported the use of a custom-built dual confocal profilometer device to measure leaf thickness across a tomato introgression line population and to map quantitative trait loci (QTL) for this trait (Coneva et al., 2017). Here, we used this device to measure leaf thickness across the *Arabidopsis thaliana* Ler × Cvi recombinant inbred line (RIL) population (Alonso-Blanco et al., 1998). Cvi is adapted to the drier, hotter, higher irradiance conditions of the Cape Verde Islands, in contrast to the Ler ecotype, which was isolated from Northern Europe, a habitat characterized by moderate temperatures and precipitation (introduction to Abarca et al., 2001). Increased leaf thickness is thus presumably an adaptive feature of Cvi plants to the abiotic stresses of their environment. To understand the genetic architecture and anatomy underlying this functional trait, we mapped QTL for leaf thickness in the Cvi RILs and found overlap between genomic regions associated with thickness and QTL for traits relating to flowering time and light-regulated growth. Through analyses of time course imaging of vegetative growth, we show that Cvi plants grow at a slower pace than Ler, supporting the idea of a trade off between the abiotic stress tolerance conferred by thick leaves and rapid growth. We also show evidence for differences in leaf morphology and endopolyploidy profiles between these ecotypes and propose a conceptual model whereby leaf thickness is patterned by the relative timing, rate, and duration of cellular events during leaf development, with the palisade mesophyll playing a central role in relaying environmental cues such as light, and elongating to contribute to leaf thickness.

## Materials and Methods

### Plant Material and Growth Conditions

Seeds for the complete set of 162 RILs between the Landsberg *erecta* (Ler-2) and Cape Verde Islands (Cvi-1) *Arabidopsis thaliana* ecotypes were obtained from The Arabidopsis Information Resource (TAIR) (Stock number: CS22000; Alonso-Blanco et al., 1998). About 10 seeds per line were plated on 0.5X MS media plates supplemented with 0.5% sucrose and stratified at 4°C for 7 days. The plates were then transferred to a growth chamber with 16-h days at 150 μmol/m^2^/s irradiance, 21°C, and 50% humidity. After the appearance of two true leaves (7 days after transfer to light), the seedlings were transferred to individual pots and moved to growth conditions (14-h days at 400 μmol/m^2^/s irradiance, 23°C and 50% humidity) at a replication of six individuals per genotype in a randomized complete block design. After approximately 17 days in the experimental condition, leaf 5 or 6 of each plant was harvested for leaf thickness, shape, and LMA measurements as detailed below. For the analysis of leaf cross-sections, flow cytometry, and time course phenotyping experiments, Ler-2 and Cvi-1 seeds (as above) were stratified for 7 days at 4°C then placed directly on soil. After 1 week, 20 seedlings of each genotype were transferred to individual pots and grown at 16-h days, 200 μmol/m^2^/s irradiance, 23°C, and 50% humidity.

### Trait Measurements

The 5th or 6th adult leaves were harvested from each RIL plant and the adaxial (upper) surfaces were scanned with a flatbed scanner to obtain raw JPG files. Each leaf was then attached on a custom-build dual confocal profilometer device (Coneva et al., 2017) and the thickness of each leaf was measured across the leaf surface at a resolution of 0.5 mm^2^. Median thickness was calculated across each leaf using values in the range 0 mm < thickness < 0.7 mm. These thickness values were used in a mixed effects linear regression model to estimate trait values for each genotype as detailed in the following section. Additionally, entire leaves were dried and their dry mass used to calculate LMA for each leaf. Leaf outline scans were processed using custom macros in Image J (Abràmoff et al., 2004) to segment individual leaves and to threshold and binarize each leaf image. Shape descriptors area, aspect ratio, roundness, circularity, and solidity (described in detail in Chitwood et al., 2013) were extracted from binary images.

### Statistical Analyses and Data Visualization

All statistical analysis and visualization was carried out using R packages (R Core Team, 2013). Trait values for QTL analyses of leaf thickness and shape traits were identified using the mixed effect linear model packages lme4 (Bates et al., 2014) and lmerTest (Kuznetsova et al., 2015) with the Ler parent as intercept, RIL genotype as a fixed effect, and tray position attributes as random effects. Only positional effects with significant variance (*p* < 0.05) were included in the final models (“tray,” “row,” “column,” **Supplementary Dataset S1**). Heritability for leaf thickness was calculated as the relative proportion of variance due to genotype. Boxplots were generated with the package ggplot2 (Wickham, 2009).

### QTL Mapping in RIL Population

For QTL mapping of leaf thickness and LMA, trait value estimates were used and for previously published traits, reported trait values were used (**Supplementary Dataset S2**). The core RIL 99 markers were downloaded from TAIR (^1^**Supplementary Dataset S3**). For each trait, the R/qtl (Broman et al., 2003) scanone function we used with the imputation method and a walking speed of 1 cM. We performed 10,000 iterations to determine genome-wide LOD (log_10_ of odds) score significance thresholds for each trait and plotted LOD value and thresholds to visualize the results for each trait. Additionally, we performed Multiple QTL Mapping (MQM) analysis (mqmscan function in R/qtl) with walking speed of 1 cM. To compute 1.5 LOD confidence intervals, we implemented makeqtl and fitqtl functions using significant scanone loci (**Supplementary Table S1**, α < 0.05, n.perm = 10000), followed by refqtl to refine the QTL regions, and finally lodint to obtain the intervals. In the case of LMA, where no significant scanone QTL were detected, confidence intervals for the top four LOD score positions are reported.

### Trait Correlations and Hierarchical Clustering

For trait correlation analyses we included all traits reported in this manuscript (leaf thickness, LMA, and shape traits) along with several sets of previously published data, including DEV (developmental), MOR (morphological), and MET (metabolic) related traits (**Supplementary Dataset S2**). Spearman correlation coefficients (rho) were calculated between each pair of traits using the rcorr function in Hmisc (Harrell et al., 2015) and *p*-values for the correlations were corrected for False Discovery Rate using the Benjamini Hochberg method (**Supplementary Dataset S4**). Hierarchical clustering (hierarchical ward.D2 algorithm) and visualization of significant correlations (*q* < 0.05) of leaf thickness and LMA were performed in the R package pheatmap (Kolde, 2015).

### Time Course Whole Plant Phenotyping

Images of 20 Ler-2 and Cvi-1 plants were taken every 2 days for 17 days (from two true leaves until bolting) with a DSLR Cannon T2i camera attached to a photography stand. Av mode was used with ISO at 800, F5.6, custom white-balance, and manual focus. Raw JPG images were downloaded, imported into Photoshop CC2014 (Adobe), and converted to LAB color space. The “A” channel (red/green) was copied and saved as a gray scale JPG image resulting in dark-gray pixels corresponding to the plant. Custom Image J (Abràmoff et al., 2004) macros were used to binarize each image and extract its total area. The slopes of individual √area vs. time plots were used to compare growth rates between genotypes. For leaf number vs. time plots, leaf number was counted manually from raw JPG images and the slopes of these lines were compared between genotypes.

### Estimation of Nuclear Size Profiles by Flow Cytometry

The 5th leaf of 3 Ler and Cvi post-flowering plants were harvested and immediately chopped in 1 mL of ice-cold buffer LB01 as in Doležel et al. (2007). The resulting fine homogenate was filtered through a 30 μm Partec CellTrics filter (5004-004-2326) and incubated with 50 μg/mL propidium iodide (Thermo Fisher, P21493) and 50 μg/mL RNase A (Qiagen, 19101) for 20 min on ice. Fluorescence scatter data was collected without gating using a BD Acuri CS6 instrument (BD Biosciences). Plots of event count as a function of fluorescence area were used to estimate the proportion of nuclei of sizes corresponding to 2C–32C in each genotype.

### Confocal Microscopy of Leaf Cross-Sections

Leaves were fixed in FAA (4% formaldehyde, 5% glacial acetic acid, 50% ethanol), vacuum infiltrated, dehydrated through an ethanol series, rehydrated to 100% water, stained in 0.002% propidium iodide (Thermo Fisher, P21493) for 2 h, dehydrated gently to 100% ethanol, and finally cleared in 100% methyl salicylate (Sigma, M6752) for 7 days. Hand-sections were visualized with a Leica SP8 laser scanning confocal microscope using white light laser excitation at 514 nm with a 20X objective.

## Results

### Multiple Loci Regulate Leaf Thickness in the Cvi Ecotype

**FIGURE 1:**
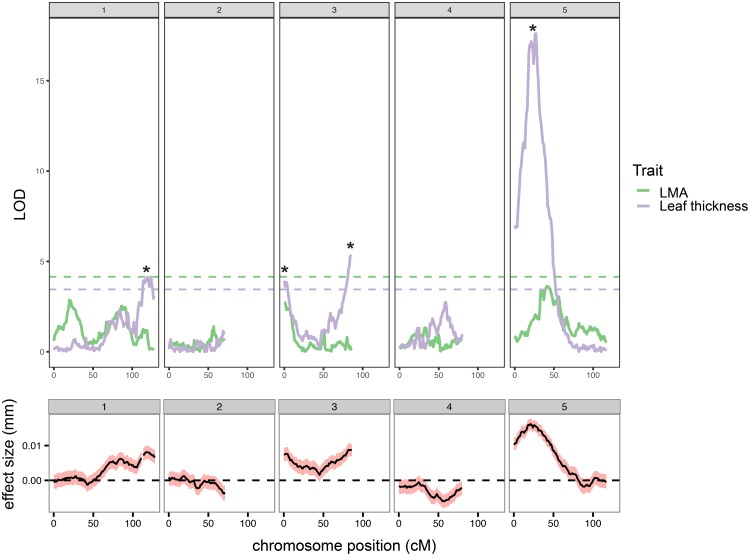
Quantitative trait loci (QTL) for leaf thickness (purple) and leaf mass per area (LMA, green) were identified using the Ler × Cvi RILs. LOD scores resulting from the scanone function in R/qtl are plotted for each trait (top). LOD significance thresholds (based on 10,000 iterations) are plotted as horizontal dashed lines. Significant leaf thickness peaks are marked with asterisks. Estimated effect size (mm leaf thickness) and associated standard error are shown in (bottom).

We used a custom-built dual confocal profilometer device to measure leaf thickness in leaf 5 or 6 of 162 Ler × Cvi RILs. We observed that leaf thickness in the Ler ecotype varies with position in the heteroblastic series, and decided on collecting measurements on the fifth and sixth leaves since they are the thickest of the adult leaves (**Supplementary Figure S1**). To determine if certain regions of the genome are associated with leaf thickness, we used publically available RIL markers to map QTL for this trait. We performed model selection in R/qtl (Broman et al., 2003) and decided to scan for all main effect QTL (scanone function). We reasoned that this approach could then be applied more universally (i.e., without introducing trait-specific dataset bias) to several other traits that we wished to compare to leaf thickness. Ultimately, we detected four loci that exceed the LOD score threshold for leaf thickness (3.48) (**Figure 1** and **Supplementary Table S1**). We also obtained LMA measurements on the same leaf and mapped QTL for this related trait. We found that the leaf thickness peaks on chromosomes 1 and 5, as well as chromosome 3@3 overlap with high LOD score LMA loci, but that the leaf thickness QTL on chromosome 3@86 is independent of LMA (**Figure 1** and **Supplementary Table S1**). Additionally, we used a maximum likelihood interval mapping approach Multiple QTL Mapping (MQM), compared the resulting LOD values to those generated by scanone and found that these two mapping approaches produce similar outputs (**Supplementary Figure S2**).

### Leaf Thickness Is Phenotypically and Genetically Associated With Delayed Flowering Time

**FIGURE 2:**
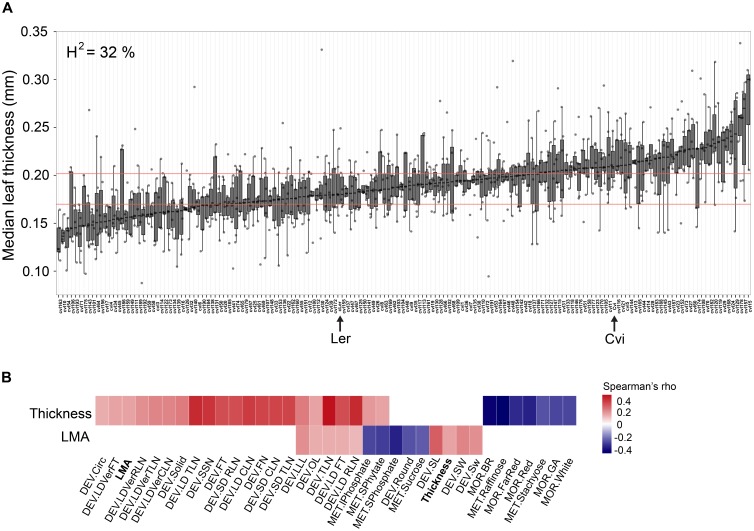
**(A)** Median leaf thickness across the RIL panel. The Ler and Cvi parents are marked with arrows. Horizontal red lines demarcate the 25th to 75th leaf thickness percentile interval for Ler. Heritability for this trait is 32%. **(B)** Significant trait correlations (Spearman’s rho) between leaf thickness (Thickness) or LMA and a suite of other traits across the Ler × Cvi RIL panel (*q* < 0.05). Traits are grouped by type: DEV, developmental; MET, metabolite; MOR, morphological (**Supplementary Datasets S1**, **S2**). Circ and Solid refer to lead shape; RLN, CLN, and TLN refer to rosette, cauline, and total leaf number, respectively; LD and SD, long and short days; Ver, vernalization; FT, flowering time; SSN, side shoot number; FN, flower number; LLL, longest leaf length; OL, ovary length; SL, seed length; SW, seed weight; BR, GA, FarRed, Red, and White, reflect hypocotyl length after treatment with brassinazole; GA, or in far red, red, or white, light conditions.

Overall, we found that leaf thickness varies widely in this population with about 32% of this variance attributable to genotype. As expected, Cvi is thicker than Ler and we observed RILs with transgressively thicker and thinner leaves than the respective parent (**Figure 2A**). To understand how leaf thickness and LMA relate to other traits in the context of developmental and/or physiological constraint, we used publically available data on the same set of Ler × Cvi RILs (summarized in Fu et al., 2009) and performed correlation analysis among traits. Neither thickness, nor LMA are significantly correlated with leaf area, indicating that thick leaves do not simply result from redistribution of leaf volume (**Supplementary Dataset S5**). We found that leaf thickness, and to some extent LMA, significantly correlate with traits associated with flowering time, total leaf number, yield, leaf shape, the abundance of several metabolites (notably several sugars and inorganic phosphate-containing compounds), and hypocotyl length in various light and growth-promoting hormone treatments (**Figure 2B** and **Supplementary Datasets S4**, **S5**) The correlations suggest that individuals with thicker leaves tend to flower later, have more leaves at flowering, and decreased hypocotyl cell elongation in response to growth-promoting stimuli. In addition, LMA, but not thickness, is also correlated with increased seed weight and size (**Figure 2B**).

**FIGURE 3:**
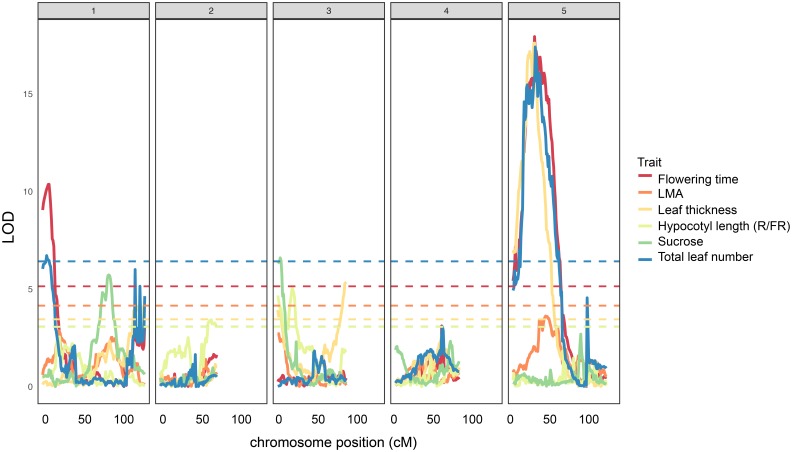
Quantitative trait loci for leaf thickness (this study), LMA (this study), flowering time, total leaf number, sucrose content, and hypocotyl length (R/FR light) in the Ler × Cvi RILs. Horizontal dashed lines show LOD significance thresholds for each trait (*p* < 0.05, 10,000 iterations).

Next, we mapped QTL for several of the traits for which we found significant correlations to leaf thickness in order to determine if there may be a genetic basis for these associations. We show that all but one of the leaf thickness QTL overlap with significant LOD peaks for one or several traits relating to flowering time, total leaf number, sucrose content, or hypocotyl length in response to R/FR light (**Figure 3**, **Supplementary Table S1**, and **Supplementary Dataset S6**). For example, leaf thickness peak chr3@3 overlaps with or is in close proximity to peaks for sucrose content (chr3@2) and hypocotyl length in R/FR light (chr3@20), while a prominent peak on chromosome 5 (chr5@22), is shared between leaf thickness, LMA, flowering time, and total leaf number. The leaf thickness peak on chromosome 1 (chr1@119) is shared with a large, albeit non-significant LOD score peak for total leaf number in this region (**Figure 3** and **Supplementary Dataset S6**). The LOD peak on chromosome 3@86 appears to be specific to leaf thickness.

### Cvi Has Reduced Leaf Growth Rate Relative to Ler

**FIGURE 4:**
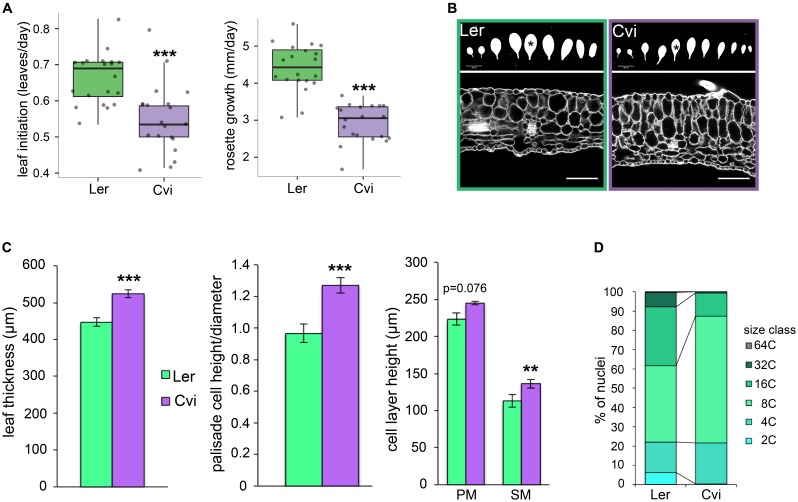
Leaf growth rate and morphology comparisons between Ler and Cvi ecotypes. **(A)** The rates of leaf initiation and projected leaf area increase were quantified from time course top-view images of 20 plants per ecotype. **(B)** Representative images of rosette leaf silhouettes of each ecotype (upper). Leaf 5 was used for all measurements and is highlighted with an asterisk. Leaf 5 cross-sections showing typical morphology for each ecotype (lower). Scale bars are 2 cm for upper panel and 100 μm for lower panel. **(C)** Leaf thickness, palisade cell dimensions, and the height of palisade and spongy mesophyll cell layers was quantified from leaf cross-section images (*n* = 12). Bars denote standard error. **(D)** Distribution of relative nuclear sizes in Ler and Cvi leaves reflecting relative endopolyploidy (*n* = 3). For **(A,C)**, ^∗∗∗^*p* < 0.001; ^∗∗^*p* < 0.01.

Given the observation that leaf thickness correlates with a suite of traits that relate to increased leaf number and delayed flowering time, as well as existing ideas that plants with thick leaves face a tradeoff between adaptation to adverse environments and growth rate, we hypothesized that Cvi plants may grow at a slower rate but make more leaves than the Ler ecotype. To test this, we imaged 20 plants of each genotype every 2 days over a period of 3 weeks (until the onset of inflorescence stem elongation) and quantified the rate of total rosette area increase and the rate of leaf initiation, as well the total number of rosette leaves. We found that, while both the rate of leaf initiation and overall growth were significantly lower for Cvi than for Ler (**Figure 4A**), Cvi makes on average 2 more leaves than Ler in long day conditions (**Supplementary Figure S3**).

### Palisade Mesophyll Cells Are Specifically Elongated in the Leaf Thickness Direction in Cvi Relative to Ler

We investigated the anatomy of Cvi and Ler leaves by comparing propidium iodide-stained cross-sections. Specifically, we quantified leaf thickness, and the dimensions of palisade and spongy mesophyll cell layers of leaf 5. In addition to having significantly thicker leaves as expected, the appearance and aspect ratio of palisade mesophyll cells in Cvi leaves is distinct from Ler (**Figures 4B,C**). Cvi palisade mesophyll cells are significantly narrower in the proximal-distal direction resulting in an increased height to width ratio and suggesting that in Cvi these cells grow anisotropically in the thickness direction. Overall, however, both palisade and spongy cell layers contribute to increased leaf thickness in Cvi (**Figure 4C**).

### Alterations in Endopolyploidy Are Associated With Leaf Thickness in Arabidopsis

To understand the cellular basis of increased leaf thickness, we performed flow cytometry on whole leaves to compare endopolyploidy profiles of Ler and Cvi.

Overall, we observed that the distribution of nuclear size classes is distinct in these ecotypes (**Figure 4D** and **Supplementary Figure S4**). The most abundant endopolyploidy level in Cvi leaves (66% of nuclei) is 8C, suggesting two endocycles, while about two thirds of Ler nuclei were approximately evenly split between the 8C and 16C sizes. The only statistically significant difference between the ecotypes was detected for the 2C size class, which is about 10 times more abundant in Ler than in Cvi leaves (6 vs. 0.5% of all detected nuclei for a given plant).

## Discussion

### Thick Leaves in the Cape Verde Islands Ecotype Are Associated With Slower Growth and Delayed Flowering Time

We measured leaf thickness, relative growth rate, and leaf initiation rate of Ler and Cvi plants. The Cvi ecotype has significantly thicker leaves and accumulates rosette biomass more slowly, at least partly due to its slower leaf initiation rate (**Figure 4A**). Taken together with significant trait correlations between leaf thickness and both leaf number and delayed flowering in a population of Ler × Cvi RILs (**Figure 2B**), these data suggest that a tradeoff may exist between leaf thickness and plant growth rate. Preferential allocation of resources to making thick leaves, which presumably increases the ability of plants to survive in arid, high light intensity environments, at the expense of vegetative growth and the timing of onset of reproductive development, is likely the mechanisms by which this trade off occurs. A recent study of leaf thickness in the desert-adapted wild tomato *S. pennellii* also found a negative correlation between leaf thickness and yield-related traits suggesting a tradeoff between investments in vegetative and reproductive development (Coneva et al., 2017). At least one study proposes that the relationship between leaf thickness and plant growth rate is linked to net CO_2_ assimilation rate, and is thus highly dependent on irradiance (Shipley, 2002). This model helps to combine several key observations including the fact that increased LMA is robustly correlated with high irradiance habitats and growth habits (Wright et al., 2004; Poorter et al., 2009), and the negative correlation between LMA and traits associated with photosynthetic productivity (Poorter et al., 2009; Edwards et al., 2014). Ultimately, species-specific investigation of the molecular basis of leaf thickness will be instrumental in determining whether this trait can be genetically uncoupled from negative associations with growth rate and yield, for example, by carefully regulating spatiotemporal gene expression.

### Genetic Loci Co-regulate Leaf Thickness, Flowering Time, and Light-Regulated Growth

We used the RIL population between Ler and Cvi ecotypes to determine QTL for leaf thickness and found four genomic regions, which are significantly associated with this trait (**Figure 1**, **Supplementary Table S1**, and **Supplementary Dataset S6**). Given the correlations between leaf thickness and flowering time (**Figure 2B**), we calculated genome-wide LOD scores for several traits relating to flowering time and plotted them alongside leaf thickness and LMA (**Figure 3** and **Supplementary Table S1**). We found that all but one leaf thickness QTL overlap with QTL for at least one trait relating to flowering time or light-regulated growth. These findings suggest that the correlations we observed between leaf thickness and flowering-time related traits (**Figure 2B**) may have a genetic basis. A study that mapped QTL for over 40,000 molecular and 139 phenotypic traits in the Ler × Cvi RILs demonstrated that six QTL hotspots accounted for most the variation across all traits, despite a high number of evenly distributed SNPs (Fu et al., 2009). These findings suggest that in Arabidopsis leaf thickness is patterned by relatively few loci with pleiotropic effects. Notably, the leaf thickness QTL on chr3@86 associated with marker HH.90L-Col appears to be specific to this trait (**Figure 3**). Further investigation of the gene(s) underlying this locus may provide insight into a morphological mechanism of leaf thickness patterning in Arabidopsis.

### Thick Cvi Leaves Have Elongated Palisade Mesophyll Cells and Distinct Endopolyploidy Profiles Compared to Ler

We compared the morphology of Cvi and Ler leaves and found striking differences between the shapes of palisade mesophyll cells in these ecotypes. Specifically, the ratio of cell height to cell width is significantly higher in Cvi leaves (**Figures 4B,C**) indicating preferential elongation of these cells in the leaf thickness direction. While both palisade and spongy mesophyll layer thickness contribute to overall leaf thickness (**Figure 4C**), the anisotropic elongation of palisade mesophyll cells in Cvi may have additional physiological implications, such as increased efficiencies of CO_2_ uptake and light distribution through the mesophyll (Brodersen et al., 2008; Niinemets et al., 2009; Brodersen and Vogelmann, 2010; Terashima et al., 2011). Moreover, the extent of palisade elongation is directly responsive to both the quality and quantity of light, an observation which suggests that high light-dependent increase in leaf thickness in Cvi (Pieruschka and Poorter, 2012) may be mediated by an increase in palisade cell height. The responsiveness of the palisade mesophyll to light is well-documented, whereby palisade cell elongation and overall leaf thickness increase in “sun leaves,” while “shade leaves” are thinner with more isodiametrically shaped cells (Esau, 1977; Terashima et al., 2005). In addition to this irradiance-dependent elongation, palisade cell length is also specifically responsive to blue-light in a number of species, including Arabidopsis (Schuerger et al., 1997; López-Juez et al., 2007; Kozuka et al., 2011; Macedo et al., 2011). Overall, these observations suggest that palisade cell elongation may be a shared morphological feature of many bifacial thick leaves, and that the environmental responsiveness of cell elongation in these cells may mediate abiotic plasticity in leaf thickness.

Aside from environmental inputs that may modulate leaf thickness, the relative timing, rate, and duration of cell proliferation and expansion in different leaf tissue types (epidermis, mesophyll, and vascular) underpins both palisade cell expansion and overall leaf thickness (Esau, 1977; Dengler, 1980; Wuyts et al., 2012; Kalve et al., 2014). In the *angustifolia* mutant, for example, alterations in the polarity of cell elongation result in narrower, thicker leaves than wild type (Tsuge et al., 1996), a phenotype reminiscent of the slender, thick leaves of the Cvi ecotype (**Figure 4B** and **Supplementary Figure S3**). Kinematic studies to capture and compare the schedule of cellular events during Ler and Cvi leaf development combined with leaf developmental modeling (Tardieu, 2003; Merks et al., 2011; Baker et al., 2015) would provide a more comprehensive view into the dynamic cellular activities that contribute to increased leaf thickness in Cvi plants.

Finally, we observed that Cvi leaves have altered endopolyploidy profiles relative to Ler leaves (**Figure 4** and **Supplementary Figure S4**). Many reports link cell size and endopolyploidy in various cell and tissue types in Arabidopsis (Melaragno et al., 1993; Roeder et al., 2010; Massonnet et al., 2011; Tsukaya, 2013). However, the extent of endoreduplication can also reflect the timing of exit from cell proliferation and the duration of cell elongation. In the absence of clear differences in cell size between Ler and Cvi leaves, their distinct endopolyploidy suggest that ecotype-specific profiles of cellular activity may accompany leaf growth and contribute to thickness patterning.

## Conclusion

We used a custom-built dual confocal profilometer device to measure leaf thickness in the Arabidopsis Ler × Cvi RIL population and found statistical support for four QTL associated with this trait. We show that three of these QTL coincide with QTL for traits related to flowering time and light-regulated growth. Along with data from trait correlation analysis, these findings support the notion that a substantial amount of the variation in leaf thickness in this population may arise pleiotropically by the genetic regulation of traits such as flowering time, which ultimately reflect the balance of resource allocation between vegetative and reproductive development. Further, we show that vegetative growth proceeds at a slower rate in the Cvi ecotype relative to Ler, supporting a tradeoff between the ability conferred by thick leaves to withstand abiotic stress and the ability to rapidly accumulate vegetative biomass. To understand the morphology of leaf thickness, we compared Cvi and Ler leaves and show evidence that Cvi palisade mesophyll cells are preferentially elongated in the leaf thickness direction, a phenotype that links leaf thickness to light-responsive palisade cell elongation. This observation provides a conceptual means by which plasticity in leaf thickness may be accomplished in response to abiotic stress, and a possible evolutionary mechanism generating natural variation in this trait. Finally, we show that Cvi leaves have a distinct endopolyploidy profile, which along with the alterations in palisade cell dimensions, suggest that mechanistically leaf thickness may be patterned by changes in the schedule of cellular events during leaf development.

## Author Contributions

VC and DC designed the research. VC conducted the research and analyzed the data. VC wrote the manuscript with contributions from DC.

## Conflict of Interest Statement

The authors declare that the research was conducted in the absence of any commercial or financial relationships that could be construed as a potential conflict of interest.

## Footnotes

1 https://www.arabidopsis.org/download/index-auto.jsp?dir=%2Fdownload_files%2FMaps%2FLer_Cvi_RIdata

## Supplementary Material

**FIGURE S1 |** Leaf thickness variation across the rosette leaf series of Ler plants (*n* = 10).

**FIGURE S2 |** Comparison of LOD scores for leaf thickness generated using R/qtl functions scanone (black), MQM (red), and MQM with BF.269C (largest LOD score locus on chromosome 5) as a co-factor (blue).

**FIGURE S3 |** Comparison of the total number of rosette leaves and the leaf 5 aspect ratio of Ler and Cvi. ^∗∗∗^*p* <0.001.

**FIGURE S4 |** Representative flow cytometry histograms of Ler and Cvi rosette leaf 5. Each peak is labeled with the presumptive nuclear size (2C–32C).

**TABLE S1 |** Summary of the position, LOD score, and 1.5 LOD interval of significant QTL peaks for leaf thickness, LMA, flowering time, total leaf number, sucrose content, and hypocotyl length (R/FR).

**DATASET S1 |** Raw trait values for leaf thickness, LMA, and leaf shape traits across the RIL panel.

**DATASET S2 |** Trait values for all traits used in trait correlation and QTL mapping analyses.

**DATASET S3 |** List of Ler × Cvi RIL SNP marker genotypes.

**DATASET S4 |** Curated list of traits included in correlation analyses and references to original data source publications.

**DATASET S5 |** Trait correlation values and FDR-corrected significance values of all pairwise trait combinations.

**DATASET S6 |** LOD scores, effect size, and standard deviation for effect size for all mapped traits (output from the scanone R/qtl function).
