# Supplementary figures and images for: Genetic and Developmental Basis for Increased Leaf Thickness in the Arabidopsis Cvi Ecotype
Source: Front Plant Sci. 2018 Mar 14;9:322. doi: 10.3389/fpls.2018.00322 (PMC5861201; doi:10.3389/fpls.2018.00322)

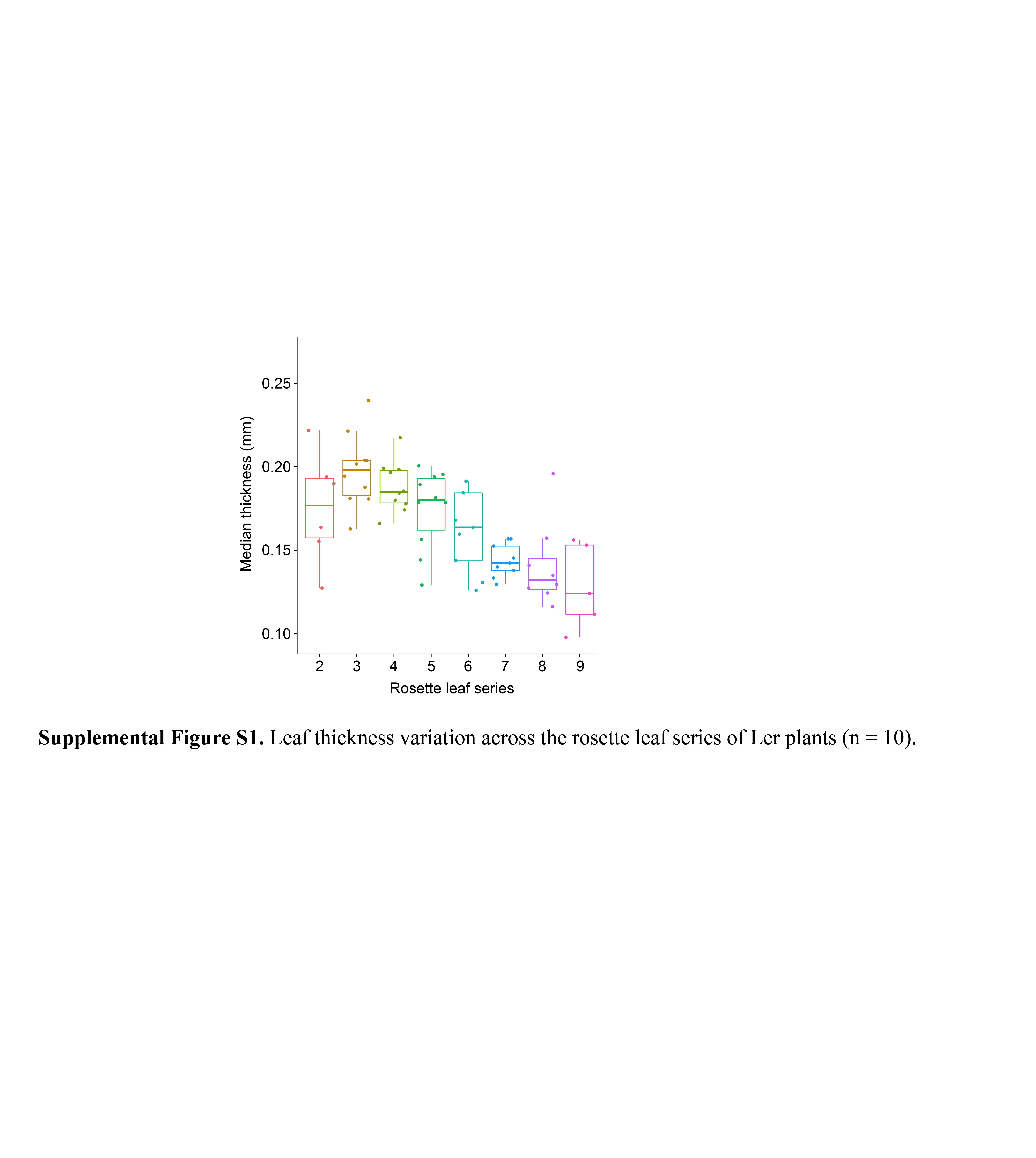

Supplement: Supplementary file 1 [file Image_1.TIF]

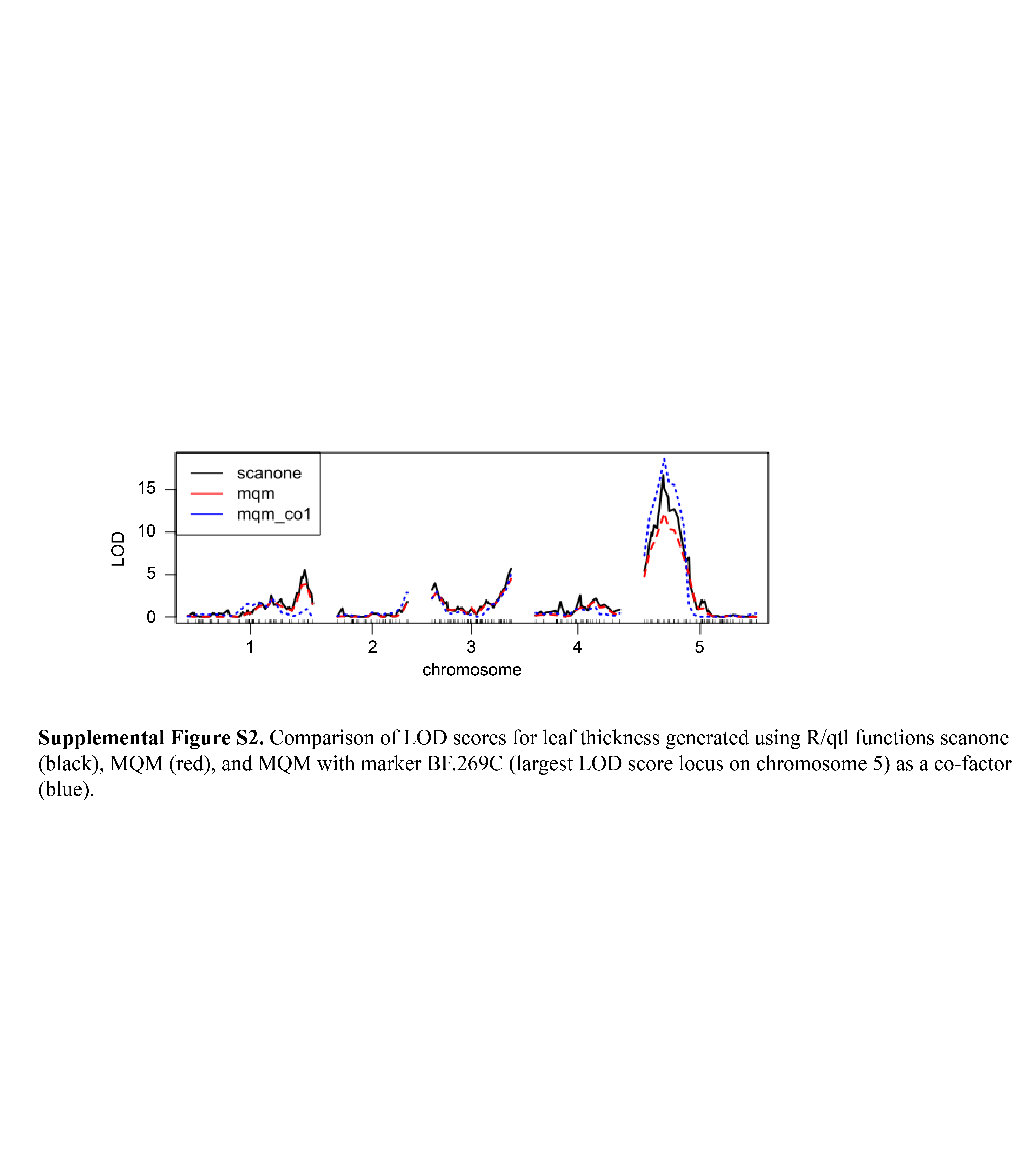

Supplement: Supplementary file 2 [file Image_2.TIF]

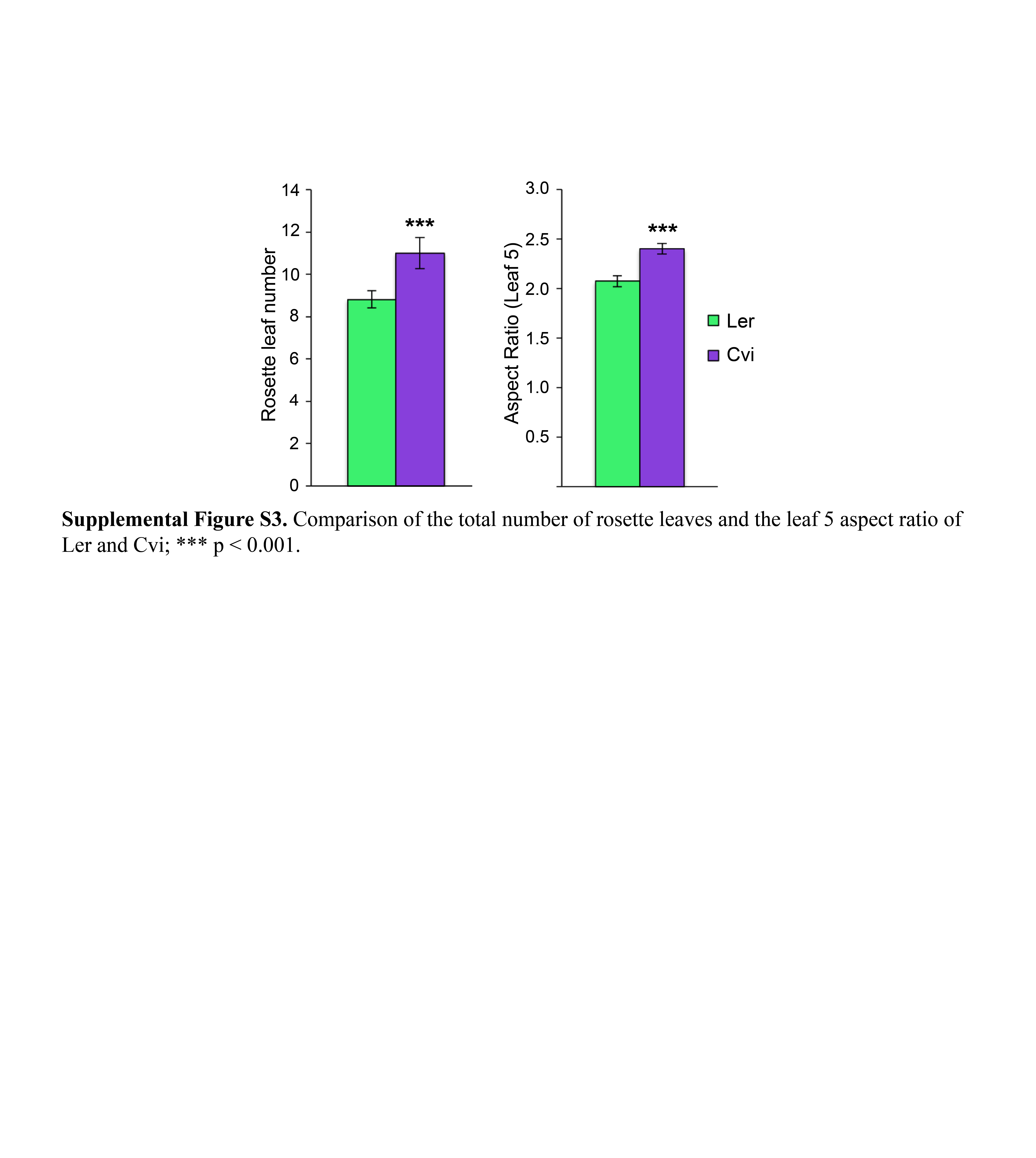

Supplement: Supplementary file 3 [file Image_3.TIF]

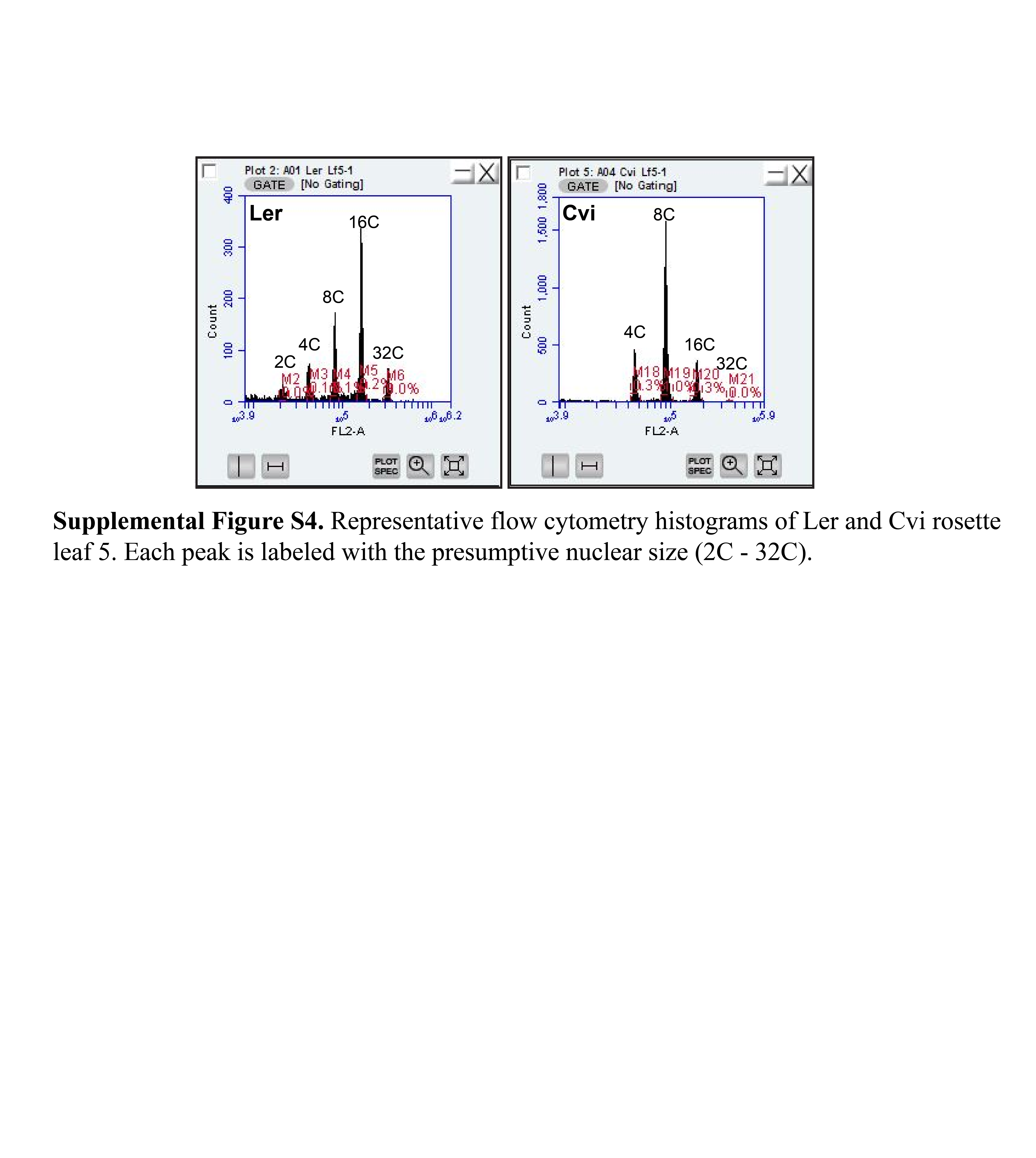

Supplement: Supplementary file 4 [file Image_4.TIF]
